# Unintentional suffocation emergencies associated with autism and common Co-occurring diagnoses

**DOI:** 10.1186/s40621-026-00723-z

**Published:** 2026-10-30

**Authors:** Ashley Blanchard, Carolyn DiGuiseppi, Caleb Ing, Matthew T. Russell, Guohua Li

**Affiliations:** 1 Department of Emergency Medicine, Columbia University Irving Medical Center, 180 Fort Washington Avenue, HP 1-104, New York, 10032 NY USA; 2 Department of Epidemiology, Colorado School of Public Health, University of Colorado Anschutz Medical Campus, 13001 East 17th Place, Campus Box B119, Aurora, 80045 CO USA; 3 Department of Anesthesiology, Columbia University Vagelos College of Physicians and Surgeons, 622 West 168th Street, New York, 10032 NY USA; 4 Department of Epidemiology, Columbia University Mailman School of Public Health, 722 West 168th Street, New York, 10032 NY USA

**Keywords:** Autism, Suffocation, Foreign body, Choking, ADHD, Alzheimer’s disease and related dementia

## Abstract

**Background:**

People with autism are at heightened risk of specific injuries and injury-related death. As autism prevalence rises, epidemiologic data describing specific injuries, such as suffocation, across the lifespan are essential for prevention efforts. We describe the risk of unintentional suffocation-related events associated with autism and common co-occurring diagnoses among people treated in United States (US) emergency departments (EDs).

**Methods:**

Using a repeated cross-sectional study design, we analyzed data from the 2016–2021 Nationwide Emergency Department Sample (NEDS), the largest US all-payer ED visit claims database. Using ICD-10-CM codes, children ≥ 1 year and adults diagnosed with autism, intellectual disability (ID), attention-deficit/hyperactivity disorder (ADHD), and Alzheimer’s disease and related dementia (ADRD) were identified and evaluated. Weighted multivariable logistic regression models were used to estimate the adjusted odds ratios (aOR) and 95% confidence intervals (CIs) of unintentional suffocation-related ED visits in persons with and without autism, ID, ADHD, and ADRD. Each model was adjusted for the other conditions, age, sex, urbanicity, and payor.

**Results:**

The 2016–2021 NEDS recorded a weighted total of 803,777,608 ED visits; of these, 1,012,210 (0.13%) were related to unintentional suffocation. Unintentional suffocation accounted for 0.35% of ED visits in patients with autism, 0.67% of ED visits in patients with a diagnosis of ID, 0.07% of ED visits in patients with a diagnosis of ADHD, and 0.34% of ED visits in patients with a diagnosis of ADRD. The odds of unintentional suffocation were increased 75% for patients with autism (aOR = 1.75; 95% CI: 1.65, 1.86), 556% for patients with ID (aOR = 6.56; 95% CI: 6.2, 6.94), 44% for patients with ADHD (aOR = 1.44; 95% CI: 1.35, 1.53), and 106% for patients with ADRD (aOR = 2.06; 95% CI: 2.01, 2.12). Across the lifespan, unintentional suffocation accounted for a larger proportion of ED visits among autistic than non-autistic patients.

**Conclusion:**

Children and adults with autism have a significantly increased odds of unintentional suffocation requiring emergency care. ID, ADHD, and ADRD, diagnoses that often co-occur with autism, also have increased odds of unintentional suffocation, with intellectual disability conferring the greatest risk.

**Supplementary information:**

The online version contains supplementary material available at https://doi.org/10.1186/s40621-026-00723-z.

## Background

Autism spectrum disorder (i.e., autism) is a common neurodevelopmental condition characterized by persistent differences in social communication and restricted, repetitive patterns of behavior, interests, or activities [1]. Rates of autism diagnoses have increased significantly, from 2.3 per 1000 in 2011 to 6.3 per 1000 in 2022 [2]. One in 31 children aged 8 years old are estimated to have autism in the US, as of 2022 [3]. Among adults, the prevalence of autism among Medicaid enrollees has increased from 4.2 per 1000 enrollees in 2011 to 9.5 per 1000 enrollees in 2019 [4]. These findings likely reflect many factors, such as the significant variation in autism diagnosis by age or a diagnostic cohort effect [5].

Individuals with autism across the age spectrum are at a significantly increased risk of injury mortality, with suffocation being the leading cause [6]. This increased risk of death was described using US national mortality data, finding a proportionate mortality ratio (PMR) of 31.93 for suffocation in people with autism, compared to the general population [6]. Suffocation incidents in individuals with autism are associated with several risk factors. Feeding challenges and differences contribute to increased suffocation risk among people with autism. Children with autism more frequently have feeding or swallowing difficulties, which may predispose them to suffocation or choking [7, 8]. Sixty percent of children and adolescents with autism have chewing problems and 76.5% have issues with rapid eating, among caregivers surveyed on feeding behaviors [9]. Wandering behavior, which is common in people with autism [10], can also increase the risk of suffocation and other accidental injuries as the person is exposed to unexpected environments.

Individuals with autism have higher rates of co-occurring diagnoses that may contribute to suffocation risk. Attention-deficit and hyperactivity disorder (ADHD) occurs in 26.2% of people with autism [11]. ADHD is associated with higher rates of unintentional injury mortality [12] and sleep-disordered breathing [13], possibly contributing to suffocation-related events. Intellectual disability (ID) occurs in 22.9% of people with autism [11], and is associated with significantly higher mortality from unintentional suffocation (due to inhalation and ingestion of objects) (HR = 26.3; 95% CI: 15.63, 44.33) [14]. Alzheimer’s disease or related dementia (ADRD) may also contribute to the risk of suffocation in people with autism. Autistic older adults are 2.6 times more likely to be diagnosed with early-onset dementia [15] and have a 20% higher adjusted risk of ADRD [16]. People with dementia are more likely to be hospitalized for suffocation (HR = 2.301, *p* < 0.001) than people without dementia [17]. Due to the cognitive decline associated with ADRD, people may develop swallowing difficulties and impaired respiratory control, contributing to an increased risk of suffocation.

Despite suffocation being a leading cause of injury death in people with autism, there is limited epidemiologic data on non-fatal suffocation-related events. Most suffocation-related events do not result in death and warrant further understanding to inform prevention efforts. Studies of ED utilization show that children and adolescents with autism more commonly present to EDs [18], providing an important environment to further understand injury, and specifically suffocation risk, among people with autism. In this study, we describe the risk and epidemiologic patterns of unintentional suffocation-related ED visits associated with autism and common co-occurring diagnoses among people treated in United States (US) EDs.

## Methods

### Data source

Data for the study is from the 2016 to 2021 Nationwide Emergency Department Sample (NEDS), the largest all-payer emergency department database in the US. NEDS is part of the Healthcare Cost and Utilization Project sponsored by the Agency for Healthcare Research and Quality [19]. It contains data from approximately 30 million ED visits each year. NEDS is constructed annually using a stratified, single-stage cluster sampling of hospital-based EDs. Sample stratification is derived from five hospital characteristics: US census region, trauma center designation, urban-rural location of the hospital, ownership, and teaching status. National estimates of all hospital-based ED visits are calculated using sampling weights for each ED visit. Data elements are compiled from state inpatient databases and state emergency department databases. Using the International Classification of Diseases, Tenth Revision, Clinical Modification (ICD-10-CM), the NEDS presents up to 35 diagnoses associated with each visit in addition to visit type, and demographic, hospital, and regional characteristics.

### Study design

To assess the risk of unintentional suffocation associated with autism as well as other conditions that commonly co-occur with autism, a repeated cross-sectional design was used. In addition to autism, the specific conditions of interest were intellectual disability (ID), attention-deficit/hyperactivity disorder (ADHD), and Alzheimer’s disease and related dementia (ADRD). These co-occurring diagnoses were identified and evaluated independent of autism status. Patients < 1 year old were excluded as the diagnosis of autism is unreliable in infants. Data from January 1, 2016 to December 31, 2021 were included in the analyses.

### Measures

Unintentional suffocation was identified using the ICD-10 injury diagnosis matrix [20]. Codes for unintentional food in the respiratory tract, gastric contents in the respiratory tract, other foreign object in the respiratory tract, and accidental asphyxiation, which captures mechanical and environmental threats to breathing (e.g., accidental smothering under a pillow, in bed linens, in furniture, or under another person’s body; suffocation in a plastic bag; accidental hanging; and entrapment in a low-oxygen environment), were included (Additional file 1 Table S1). Non-accidental suffocation, such as assault, self-harm, and undetermined intent, was excluded from the analyses. Autism was identified using ICD10 code F84.0, ADHD was identified using ICD10 code F90, ID was identified using ICD10 codes F70–F79, and ADRD was identified using codes G30, F01, and F03. Demographic variables included age, sex, region of emergency department, and insurance payor (Medicaid, Medicare, private insurance, self-pay, and other). Before 2019, race and ethnicity data were not available [19].

### Statistical analyses

The weighted total number of ED visits and unintentional suffocation-related ED visits were calculated according to age, sex, urbanicity, payor status, autism, ID, ADHD, and ADRD using the Rao-Scott approach [21]. Adjusted estimated odds ratios (OR) and 95% confidence intervals (95% CIs) were calculated using multivariable weighted logistic regression modeling, comparing unintentional suffocation among people with and without autism and ADHD, ID, and ADRD. Models were adjusted for age, sex, urbanicity, payor, ID, ADHD, and ADRD. Weighted frequencies of suffocation type by autism status and the proportion of overall ED visits for suffocation by autism status by age group were analyzed. Sensitivity analysis restricting the data to 2019–2021 was completed to determine the influence of race and ethnicity on results.

## Results

**Fig. 1:**
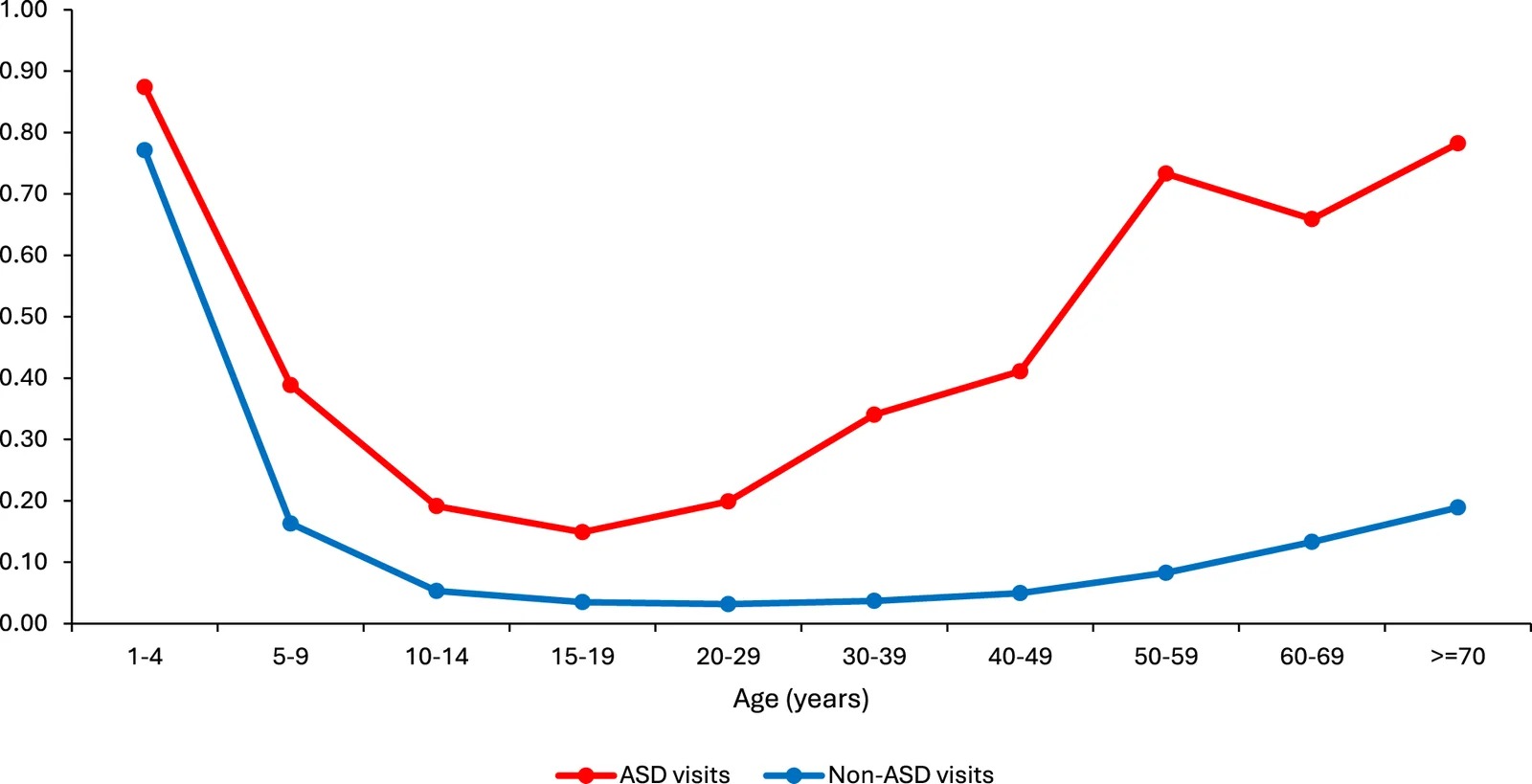
Proportion of emergency department (ED) visits for unintentional suffocation by age group and autism spectrum disorder (ASD) status in patients aged 1 year and above, weighted data from the Nationwide Emergency Department Sample 2016–2021

From 2016 to 2021, NEDS reported 187,847,994 ED visits; of these, 234,987 (0.13%) were for unintentional suffocation among people ≥ 1 year of age (Additional file 1 Table S2). Weighted analysis revealed 803,777,608 ED visits; of these, 1,012,210 (0.13%) were for unintentional suffocation among people ≥ 1 year of age (Table 1). The prevalence of ED visits for unintentional suffocation varied by demographic characteristics (Table 1). Across the lifespan, the prevalence of unintentional suffocation among ED visits by age group was highest among children ages 1–4 years (0.77%) and older adults * ≥ *70 years (0.19%). Children ages 1–4 years had the highest odds of unintentional suffocation (aOR = 11.44; 95% CI: 10.97, 11.94), compared to children ages 10–15 years. The prevalence of unintentional suffocation among ED visits was higher among people with autism across all age groups. The prevalence of unintentional suffocation increased at the youngest and oldest age groups, but the increase was less pronounced in people without autism (Fig. 1). The prevalence of unintentional suffocation was slightly lower among females compared to males (0.11% vs 0.14%; *p* < 0.001). People with private insurance had a very small increased odds of unintentional suffocation (aOR = 1.05; 95% CI: 1.03, 1.07), while people with self-pay had a slightly lower odds of unintentional suffocation (aOR = 0.85; 95% CI: 0.82, 0.87), in comparison to people with Medicare or Medicaid.

**Table 1:** Weighted frequencies and estimated odds ratios (OR) and 95% confidence intervals (CI) of unintentional suffocation according to demographic and clinical characteristics in patients aged 1 year and above, weighted data from the Nationwide Emergency Department Sample 2016–2021

Characteristic	Number of ED visits	Number of ED visits for suffocation (%)	Crude OR (95% CI)	p value	Adjusted* OR (95% CI)	p value
**All**	803,777,608	1,012,210 (0.13)				
**Age**						
1–4	45,363,363	349,993 (0.77)	11.09 (10.62, 11.58)	<0.0001	11.44 (10.97, 11.94)	<0.0001
5–9	32,487,966	53,769 (0.17)	2.4 (2.31, 2.5)	<0.0001	2.44 (2.34, 2.54)	<0.0001
10–14	29,611,265	16,184 (0.05)	1.00		1.00	<0.0001
15–19	44,492,507	15,861 (0.04)	0.83 (0.79, 0.87)	<0.0001	0.85 (0.81, 0.89)	<0.0001
20–29	122,539,309	39,416 (0.03)	0.75 (0.72, 0.79)	<0.0001	0.79 (0.75, 0.82)	<0.0001
30–39	113,994,107	42,394 (0.04)	0.77 (0.74, 0.81)	<0.0001	0.81 (0.78, 0.85)	<0.0001
40–49	95,864,216	47,718 (0.05)	0.86 (0.82, 0.9)	<0.0001	0.89 (0.85, 0.93)	<0.0001
50–59	101,921,206	84,609 (0.08)	1.26 (1.21, 1.32)	<0.0001	1.28 (1.23, 1.34)	<0.0001
60–69	88,106,532	117,428 (0.13)	1.93 (1.84, 2.03)	<0.0001	1.94 (1.85, 2.03)	<0.0001
≥70	129,397,136	244,839 (0.19)	2.71 (2.58, 2.84)	<0.0001	2.53 (2.41, 2.65)	<0.0001
**Sex**						
Female	443,762,028	501,175 (0.11)	0.81 (0.8, 0.82)	<0.0001	0.91 (0.9, 0.93)	<0.0001
Male	360,015,580	511,037 (0.14)	1.00		1.00	
**Urbanicity**						
Urban	663,537,205	838,634 (0.13)	1.03 (0.98, 1.07)	0.2371	1.03 (0.99, 1.07)	0.1427
Suburban	84,044,702	103,661 (0.12)	1 (0.96, 1.04)	0.9461	1.02 (0.98, 1.06)	0.3337
Rural	56,195,702	69,916 (0.12)	1.00		1.00	
**Payor**						
Medicare/Medicaid	442,864,067	667,973 (0.15)	1.00		1.00	
Medicaid	243,012,568	330,400 (0.14)				
Medicare	199,851,499	337,574 (0.17)				
Private insurance	231,040,201	256,540 (0.11)	0.76 (0.74, 0.77)	<0.0001	1.05 (1.03, 1.07)	<0.0001
Self-pay	93,058,422	55,507 (0.06)	0.47 (0.46, 0.49)	<0.0001	0.85 (0.82, 0.87)	<0.0001
Other	36,814,918	32,192 (0.09)	0.71 (0.68, 0.74)	<0.0001	1.04 (1, 1.08)	0.0321
**Autism spectrum disorder**						
Yes	1,702,941	5,917 (0.35)	2.77 (2.6, 2.95)	<0.0001	1.75 (1.65, 1.86)	<0.0001
No	802,074,666	1,006,294 (0.13)	1.00		1.00	
**Intellectual disability**						
Yes	1,594,847	10,632 (0.67)	5.09 (4.8, 5.4)	<0.0001	6.56 (6.2, 6.94)	<0.0001
No	802,182,761	1,001,580 (0.12)	1.00		1.00	
**Attention-deficit hyperactivity disorder**						
Yes	5,140,847	3,840 (0.07)	0.93 (0.87, 0.99)	0.022	1.44 (1.35, 1.53)	<0.0001
No	798,636,761	1,008,372 (0.13)	1.00		1.00	
**Alzheimer’s disease and related dementia**						
Yes	16,166,670	55,167 (0.34)	2.61 (2.52, 2.7)	<0.0001	2.06 (2.01, 2.12)	<0.0001
No	787,610,938	957,045 (0.12)	1.00		1.00	

*Adjusted for age, sex, urbanicity, payor, autism spectrum disorder, intellectual disability, attention-deficit hyperactivity disorder, and Alzheimer’s disease and related dementia

Unintentional suffocation accounted for 0.35% of ED visits among people with autism, 0.67% of ED visits among people with ID, 0.07% of ED visits among people with ADHD, and 0.34% of ED visits among people with ADRD. When adjusting for age, sex, urbanicity, payor, ID, ADHD, and ADRD, people with autism had a significantly increased odds of unintentional suffocation (aOR = 1.75; 95% CI: 1.65, 1.86), compared to people without autism. Relative to patients without ADHD, the presence of ADHD was associated with an increased odds of unintentional suffocation (aOR = 1.44; 95% CI: 1.35, 1.53). ADRD had the second largest increased odds of unintentional suffocation (aOR = 2.06; 95% CI: 2.01, 2.12), and ID had the largest increased odds of unintentional suffocation (aOR = 6.56; 95% CI: 6.2, 6.94).

**Fig. 2:**
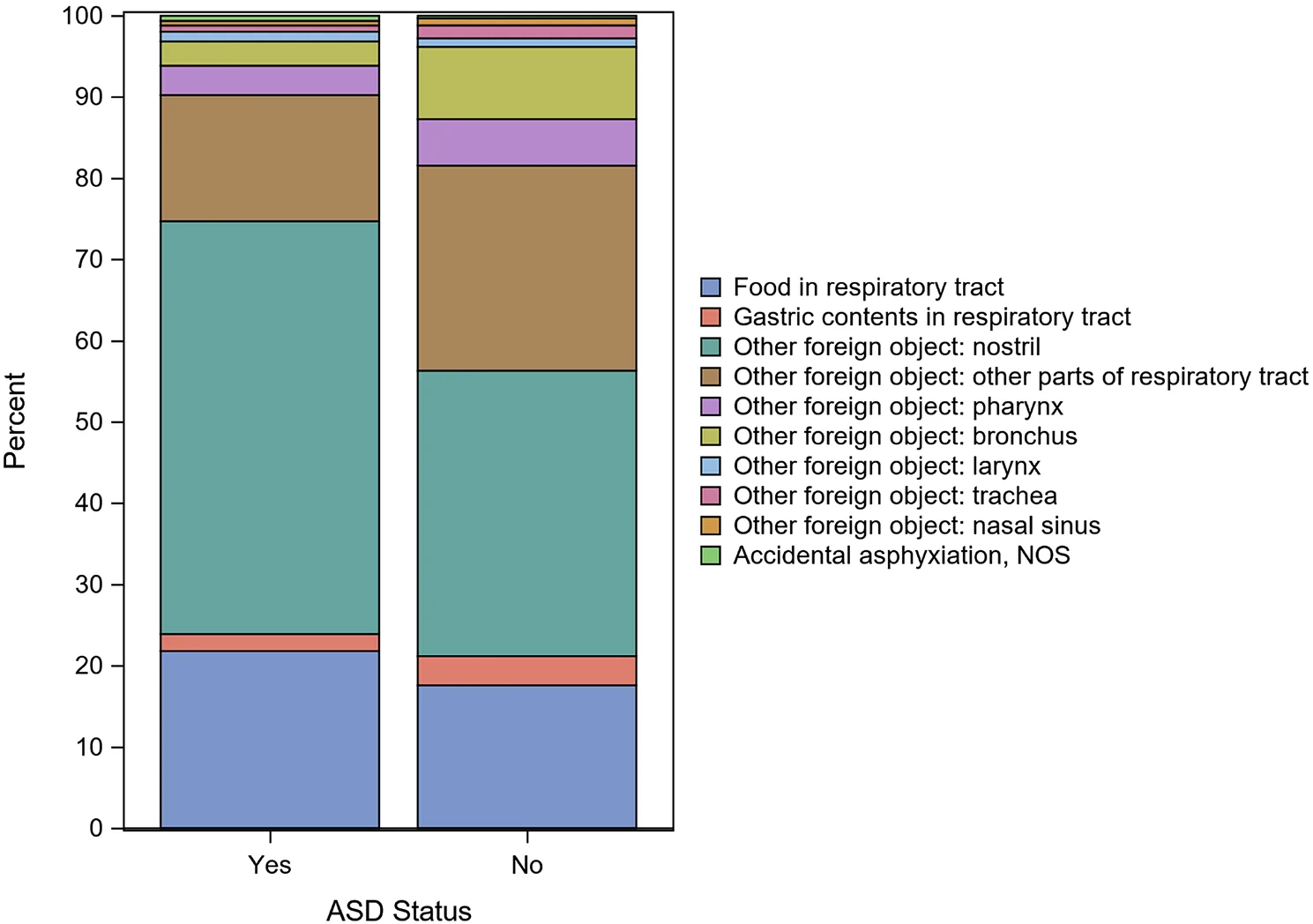
Percentage distributions of types of emergency department-related visits for suffocation by autism spectrum disorder (ASD) status in patients aged 1 year and above, weighted data from the Nationwide Emergency Department Sample 2016–2021

Percentage distributions by type of suffocation were similar between people with and without autism (Fig. 2). Of the 5,917 unintentional suffocation emergencies among people with autism, the most common type was “other foreign object in the respiratory tract” (75.5%). Of the specific locations for “other foreign object in the respiratory tract”, nostril was the most common location of suffocation-related events among people with (50.8%) and without (35.1%) autism. Choking or suffocation incidents related to “food in the respiratory tract” accounted for 21.8% of unintentional suffocation emergencies among people with autism and 17.6% of unintentional suffocation emergencies among people without autism. Among children ages 1–4 years, a larger proportion of unintentional suffocation emergencies were related to a foreign object in the nostril (Additional file 1 Table S3).

Disposition from the ED for unintentional suffocation is summarized in Table 2. Of the 1,006,294 unintentional suffocation-related ED visits among people without autism, 59.4% were discharged home, 39.3% were admitted to the hospital, and 0.59% died. Of the 5,917 unintentional suffocation-related ED visits among people with autism, 76.0% were discharged, 23.0% were admitted to the hospital, and 0.65% died.

**Table 2:** Disposition of unintentional suffocation emergencies by autism spectrum disorder (ASD) status in patients aged 1 year and above, weighted data from the Nationwide Emergency Department Sample 2016–2021

	Without ASD	With ASD
*Disposition from ED*	*n (%)*	*n (%)*
Admitted to hospital	395660 (39.32)	1359 (22.97)
Died	5967 (0.59)	38 (0.65)
Discharged	597753 (59.40)	4497 (76.01)
Left AMA	6913 (0.69)	21 (0.36)
Total	1006294 (100)	5916 (100)

Abbreviation: AMA = against medical advice

To examine the potential influence of race and ethnicity, data were restricted to 2019–2021, when race and ethnicity became available in NEDS. The odds of suffocation-related ED visits among people with autism, adjusted for race and ethnicity (in addition to age, sex, urbanicity, payor, ID, ADHD, and ADRD) in comparison to people without autism (aOR = 1.60; 95% CI: 1.48, 1.74) were similar to the complete 6-year analysis excluding race and ethnicity (aOR = 1.75; 95% CI: 1.65, 1.86) (Additional file 1 Table S4).

## Discussion

This national study examined the epidemiologic patterns and risks of unintentional suffocation requiring emergency care among people with autism across the lifespan. The odds of unintentional suffocation are significantly higher among people with autism, after accounting for differences in commonly co-occurring conditions, consistent with prior mortality data showing suffocation as the leading cause of mortality among people with autism [6]. This study extends these mortality findings by characterizing suffocation events requiring emergency care that are mostly non-fatal, providing insight into the broader spectrum of suffocation-related morbidity in this vulnerable population.

The prevalence of unintentional suffocation among ED visits was higher among people with autism across all age groups, ≥1 year of age. While people with and without autism had increasing proportions of ED visits for unintentional suffocation with increasing age, the increase with age was much larger in people with autism. This is, in part, due to the cohort effect in autism diagnosis, causing older adult cohorts to have more severe symptoms than younger cohorts, predisposing them to more oral-motor symptoms and suffocation [5]. The higher proportion of suffocation-related ED visits among autistic adults in the older age groups (e.g., 50–59 years) should be interpreted with caution, as the number of autistic adults in these older strata is small and yields less stable estimates. This upward pattern is consistent with the autism diagnostic cohort effect described above, as well as with age-related increases in dysphagia and neurodegenerative comorbidity that heighten suffocation risk in later life.

The type of suffocation was similar between the autistic and non-autistic population with “other foreign object in the respiratory tract” being more common than “food in the respiratory tract”. People with autism more commonly presented to the ED with a nasal foreign object in the respiratory tract, consistent with prior literature [22]. Nasal foreign body is a subcategory of foreign body in the respiratory tract. Nasal foreign object codes were included, as they fall under the category of foreign object in the respiratory tract, which may cause suffocation. Nasal foreign body may represent a less severe event, though further details about the circumstances of the event are not available in the NEDS data system. ED visits for unintentional suffocation among people with autism less commonly resulted in an admission to the hospital, in comparison to people without autism. This lower overall admission rate in the autism group is potentially confounded by age, as people with autism in this sample were considerably younger than those without autism. This may also reflect less severe ED presentations among people with autism or the higher proportion of nasal foreign objects in the respiratory tract. As with many acute presentations in people with autism, limitations in communication may cause greater escalations in choking events, resulting in more ED visits for self-resolving choking or gagging episodes. The oromotor challenges in people with autism may contribute to more frequent choking episodes that self-resolve but still result in an ED visit.

People with ADHD were also shown to have increased odds of unintentional suffocation-related ED visits, though slightly lower odds than those of people with autism. While it is known that children with ADHD have higher rates of ED visits for physical injuries compared to controls [18], the increased risk of suffocation-related ED visits has not previously been described in the literature. People with ADHD have increased impulsivity and risk-taking behaviors, potentially creating increased environmental risk for suffocation. The odds of unintentional suffocation-related ED visits were also higher among people with ADRD, consistent with the known risk of suffocation. This further supports prior literature demonstrating the significantly increased risk of hospitalization for suffocation among adults with ADRD (HR = 2.301, *p* < 0.001) [17] due to known dysphagia and aspiration risk associated with ADRD. ID was associated with the highest odds of unintentional suffocation-related ED visits, further supporting the literature on the association between ID and suffocation risk [14, 23]. A recent study using data from the Autism and Developmental Disabilities Monitoring Network described significantly increased mortality from choking (HR = 26.3; 95% CI: 15.63, 44.33) among youth and young adults with ID [14]. Our study describes a similarly increased risk, expanding on the current literature to include older adults and non-fatal events.

Patterns of suffocation-related ED visits also varied by insurance status. Relative to patients insured by Medicare or Medicaid, patients with private insurance had marginally higher adjusted odds of a suffocation-related ED visit (aOR = 1.05; 95% CI: 1.03, 1.07), whereas self-pay patients had lower adjusted odds (aOR = 0.85; 95% CI: 0.82, 0.87). Although one might expect higher rates of suffocation-related visits, admissions, and deaths among publicly insured and uninsured populations, the lower observed rate among self-pay patients may reflect barriers to accessing emergency care rather than a lower burden of suffocation injury. Because young children with nasal foreign bodies account for a large share of suffocation-related visits, differences in supervision, care-seeking behavior, and socioeconomic circumstances across insurance groups may also contribute to the observed patterns. These findings should therefore be interpreted as likely reflecting differences in access to and utilization of emergency care rather than definitive differences in underlying risk.

Understanding the epidemiologic association between autism and suffocation-related injuries can inform targeted prevention strategies, improve clinical awareness among emergency medicine providers, and guide public health initiatives aimed at reducing injury burden in this vulnerable population. These findings should increase awareness among physicians and other healthcare providers of the risk of suffocation among people with autism, leading to increased parental education to both prevent and mitigate choking and suffocation-related events. Parents should be educated on harm prevention strategies, such as appropriate food offerings, the Heimlich maneuver, and speech and swallow services. Further research should investigate specific circumstances such as detailed mechanisms, environmental factors, and preceding events that may be more specific to people with autism.

## Limitations

Our study has important limitations. Unintentional suffocation-related ED visits capture a spectrum of choking, gagging, asphyxiation and smothering. These types of events can be transient and often hard to characterize or describe retrospectively. Specifically nasal foreign body in the respiratory tract likely represents the spectrum of transient self-resolving events to fatal suffocation. We were able to assess ED disposition, but our data does not further characterize ED interventions or acuity of the ED visit. Inherent to a large-scale data analysis and ICD-10-CM codes, there is also some degree of misclassification of events and autism diagnosis, particularly among younger children. Ascertainment of intent also presents a challenge, particularly among people with autism who commonly have limitations in communication and are predisposed to self-harm and at increased risk of child maltreatment [6, 24–27]. The NEDS only captures suffocation events that result in emergency department presentation, excluding presentations treated in other healthcare settings or those that do not receive medical attention, which may lead to underestimation of the true burden of suffocation injuries. Finally, we did not examine the potential contribution of other conditions commonly co-occurring with autism, such as seizure disorders, which may independently influence suffocation risk and warrant investigation in future studies.

## Conclusions

This nationally representative sample of ED visits describes the prevalence of suffocation-related ED visits among people with autism across the lifespan. People with autism, ADHD, ID, and ADRD are at significantly increased odds of unintentional suffocation-related ED visits, with people with ID having the greatest odds of unintentional suffocation-related ED visits. Children ages 1–4 years and adults ages 70 years and older accounted for the largest proportions of unintentional suffocation-related ED visits among people with and without autism. Most commonly, unintentional suffocation-related ED visits were from other foreign objects in the nose and resulted in a discharge from the ED. Future studies should investigate the mechanisms of suffocation-related events among people with autism and explore interventions to prevent unintentional suffocation-related events. Understanding the increased odds of suffocation among people with autism should increase provider and family member awareness of primary and secondary prevention methods, such as CPR training and oromotor therapy.

## Electronic supplementary material

Below is the link to the electronic supplementary material.

Supplementary Material 1

## Data Availability

The datasets generated and analyzed for this study are available in the NEDS repository, https://www.hcup-us.ahrq.gov/db/nation/neds/nedsdbdocumentation.jsp.

## Abbreviations

aOR: Adjusted odds ratio
CI: Confidence interval
ED: Emergency department
ICD-10-CM: International Classification of Diseases, Tenth Revision, Clinical Modification
ID: Intellectual disability
ADRD: Alzheimer’s disease and related dementia
ADHD: Attention deficit and hyperactivity disorder
NEDS: Nationwide Emergency Department Sample
US: United States
