# Supplementary material for: Unintentional suffocation emergencies associated with autism and common Co-occurring diagnoses
Source: Inj Epidemiol. 2026 Oct 30;13(Suppl 1):69. doi: 10.1186/s40621-026-00723-z (PMC13726090; doi:10.1186/s40621-026-00723-z)
Supplement: Supplementary file 1 [file 40621_2026_723_MOESM1_ESM.docx]

| **Additional file 1: Supplementary Tables** **Table S1.** ICD-10-CM codes for suffocation. **Suffocation** \| **Suffocation Type** \| **Code**; Unintentional suffocation \| Accidental \| T71111, T71121, T71131, T71141, T71151, T71161, T71191, T71221, T71231; \| Food in respiratory tract \| T1722, T1732, T1742, T1752, T1782, T1792; \| Gastric contents in respiratory tract \| T1721, T1731, T1741, T1751, T1781, T1791; \| Other foreign object in respiratory tract \| All T17 codes except T1721, T1722, T1731, T1732, T1741, T1742, T1751, T1752, T1781, T1782, T1791, and T1792; Intentional suffocation \| Assault \| T71113, T71123, T71133, T71143, T71153, T71163, T71193, T71223, T71233; \| Self-harm \| T71112, T71122, T71132, T71152, T71162, T71192, T71222, T71232; \| Undetermined \| All T71 codes except T71112, T71113, T71122, T71123, T71132, T71133, T1143, T71152, T71153, T71162, T71163, T71192, T71193, T71222, T71223, T71232, and T71233 **Table S2.** Unweighted frequencies and estimated odds ratios (OR) and 95% confidence intervals (CI) of suffocation-related emergency department (ED) visits according to demographic and clinical characteristics in patients aged 1 year and above, unweighted data from the Nationwide Emergency Department Sample 2016–2021. \| \| \| \| \| \| \| \| \| \| \|; **Characteristic** \| **Number of ED visits** \| **Number of ED visits for suffocation (%)** \| **Crude OR** **(95% CI)** \| **p value** \| **Adjusted* OR** **(95% CI)** \| **p value** \| \| \| \| \|; **All** \| 187,847,994 \| 234,987(0.13) \| \| \| \| \| \| \| \| \|; **Age** \| \| \| \| \| \| \| \| \| \| \|; 1-4 \| 10,575,479 \| 81,311 (0.77) \| 11.21 (10.89, 11.54) \| <.0001 \| 11.57 (11.23, 11.91) \| <.0001 \| \| \| \| \|; 5-9 \| 7,580,537 \| 12,441 (0.16) \| 2.41 (2.34, 2.5) \| <.0001 \| 2.45 (2.37, 2.53) \| <.0001 \| \| \| \| \|; 10-14 \| 6,885,079 \| 3,716 (0.05) \| 1.00 \| \| 1.00 \| \| \| \| \| \|; 15-19 \| 10,374,514 \| 3,660 (0.04) \| 0.83 (0.8, 0.87) \| <.0001 \| 0.85 (0.82, 0.88) \| <.0001 \| \| \| \| \|; 20-29 \| 28,827,842 \| 9,224 (0.03) \| 0.76 (0.73, 0.78) \| <.0001 \| 0.79 (0.77, 0.82) \| <.0001 \| \| \| \| \|; 30-39 \| 26,789,370 \| 9,887 (0.04) \| 0.78 (0.76, 0.81) \| <.0001 \| 0.82 (0.79, 0.85) \| <.0001 \| \| \| \| \|; 40-49 \| 22,499,851 \| 11,149 (0.05) \| 0.87 (0.84, 0.9) \| <.0001 \| 0.9 (0.87, 0.93) \| <.0001 \| \| \| \| \|; 50-59 \| 23,836,850 \| 19,653 (0.08) \| 1.27 (1.23, 1.31) \| <.0001 \| 1.3 (1.26, 1.34) \| <.0001 \| \| \| \| \|; 60-69 \| 20,524,639 \| 27,312 (0.13) \| 1.96 (1.9, 2.02) \| <.0001 \| 1.96 (1.91, 2.03) \| <.0001 \| \| \| \| \|; >=70 \| 29,953,833 \| 56,634 (0.19) \| 2.75 (2.67, 2.83) \| <.0001 \| 2.56 (2.49, 2.64) \| <.0001 \| \| \| \| \|; **Sex** \| \| \| \| \| \| \| \| \| \| \|; Female \| 103,818,344 \| 116,282 (0.11) \| 0.8 (0.8, 0.81) \| <.0001 \| 0.91 (0.9, 0.92) \| <.0001 \| \| \| \| \|; Male \| 84,029,650 \| 118,705 (0.14) \| 1.00 \| \| 1.00 \| \| \| \| \| \|; **Urbanicity** \| \| \| \| \| \| \| \| \| \| \|; Urban \| 157,574,242 \| 197,499 (0.13) \| 1.01 (1, 1.03) \| 0.1048 \| 1.02 (1.00, 1.03) \| 0.0362 \| \| \| \| \|; Suburban \| 18,147,667 \| 22,322 (0.12) \| 0.99 (0.97, 1.01) \| 0.3697 \| 1.01 (0.99, 1.03) \| 0.311 \| \| \| \| \|; Rural \| 12,126,085 \| 15,166 (0.13) \| 1.00 \| \| 1.00 \| \| \| \| \| \|; **Payor** \| \| \| \| \| \| \| \| \| \| \|; Medicare/Medicaid \| 103,374,744 \| 155,159 (0.15) \| 1.00 \| \| 1.00 \| \| \| \| \| \|; Medicaid \| 57,116,138 \| 77,051 (0.13) \| \| \| \| \| \| \| \| \|; Medicare \| 46,258,606 \| 78,108 (0.17) \| \| \| \| \| \| \| \| \|; Private insurance \| 53,701,152 \| 59,280 (0.11) \| 0.75 (0.75, 0.76) \| <.0001 \| 1.05 (1.04, 1.06) \| <.0001 \| \| \| \| \|; Self-pay \| 22,250,004 \| 13,113 (0.06) \| 0.47 (0.46, 0.48) \| <.0001 \| 0.85 (0.83, 0.86) \| <.0001 \| \| \| \| \|; Other \| 8,522,094 \| 7,435 (0.09) \| 0.71 (0.7, 0.72) \| <.0001 \| 1.05 (1.03, 1.07) \| <.0001 \| \| \| \| \|; **Year** \| \| \| \| \| \| \| \| \| \| \|; 2016 \| 31,713,317 \| 39,490 (0.12) \| \| \| \| \| \| \| \| \|; 2017 \| 32,602,746 \| 41,291 (0.13) \| \| \| \| \| \| \| \| \|; 2018 \| 34,716,808 \| 43,744 (0.13) \| \| \| \| \| \| \| \| \|; 2019 \| 32,218,028 \| 41,173 (0.13) \| \| \| \| \| \| \| \| \|; 2020 \| 27,312,186 \| 35,220 (0.13) \| \| \| \| \| \| \| \| \|; 2021 \| 29,284,909 \| 34,069 (0.12) \| \| \| \| \| \| \| \| \|; **Autism spectrum disorder** \| \| \| \| \| \| \| \| \| \| \|; Yes \| 393,374 \| 1,388 (0.35) \| 2.83 (2.69, 2.98) \| <.0001 \| 1.79 (1.7, 1.88) \| <.0001 \| \| \| \| \|; No \| 187,454,620 \| 233,599 (0.12) \| 1.00 \| \| 1.00 \| \| \| \| \| \|; **Intellectual disability** \| \| \| \| \| \| \| \| \| \| \|; Yes \| 362,143 \| 2,386 (0.66) \| 5.07 (4.87, 5.28) \| <.0001 \| 6.51 (6.25, 6.78) \| <.0001 \| \| \| \| \|; No \| 187,485,851 \| 232,601 (0.12) \| 1.00 \| \| 1.00 \| <.0001 \| \| \| \| \|; **Attention-deficit hyperactivity disorder** \| \| \| \| \| \| \| \| \| \| \|; Yes \| 1,174,042 \| 879 (0.07) \| 0.94 (0.89, 0.99) \| 0.0157 \| 1.46 (1.38, 1.53) \| <.0001 \| \| \| \| \|; No \| 186,673,952 \| 234,108 (0.13) \| 1.00 \| \| 1.00 \| \| \| \| \| \|; **Alzheimer's disease and related dementia** \| \| \| \| \| \| \| \| \| \| \|; Yes \| 861,545 \| 2,968 (0.34) \| 2.61 (2.57, 2.66) \| <.0001 \| 2.05 (2.02, 2.09) \| <.0001 \| \| \| \| \|; No \| 186,986,449 \| 232,019 (0.12) \| 1.00 \| \| 1.00 \| \| \| \| \| \|; *Adjusted for age, sex, urbanicity, payor, autism spectrum disorder, intellectual disability, attention-deficit hyperactivity disorder, and Alzheimer’s disease and related dementia. \| \| \| \| \| \| \| \| \| \| \| | | | | | | |  |
| --- | --- | --- | --- | --- | --- | --- | --- |
| **Table S3.** Percentage distributions of types of emergency department visits for suffocation by autism spectrum disorder (ASD) status in patients aged 1-4 years, weighted data from the Nationwide Emergency Department Sample 2016–2021. \| **ASD visits** \| \| **Non-ASD visits** \|; **Total** \| 1,706 \| 100.00% \| 348,288 \| 100.00%; **Suffocation type** \| Number \| Percent \| Number \| Percent; Accidental asphyxiation due to mechanical threat to breathing or systemic oxygen deficiency \| 4 \| 0.23% \| 505 \| 0.14%; Food in respiratory tract \| 79 \| 4.65% \| 18,208 \| 5.23%; Gastric contents in respiratory tract \| 12 \| 0.71% \| 1,142 \| 0.33%; Other foreign object in respiratory tract \| \| \| \|; bronchus \| 8 \| 0.48% \| 1,311 \| 0.38%; larynx \| 16 \| 0.93% \| 2,319 \| 0.67%; nasal sinus \| 12 \| 0.68% \| 6,492 \| 1.86%; nostril \| 1,475 \| 86.43% \| 297,608 \| 85.45%; other parts of respiratory tract \| 58 \| 3.43% \| 13,753 \| 3.95%; pharynx \| 42 \| 2.44% \| 6,243 \| 1.79%; trachea \| 0 \| 0.00% \| 706 \| 0.20% **Table S4.** Weighted frequencies and estimated odds ratios (OR) and 95% confidence intervals (CI) of suffocation-related emergency department (ED) visits according to demographic and clinical characteristics in patients aged 1 year and above, weighted data from the Nationwide Emergency Department Sample 2019–2021. | | | | | | |  |
|  |  |  |  |  |  |  |  |
| **Characteristic** | **Number of ED visits** | **Number of ED visits for suffocation (%)** | **Crude OR (95% CI)** | **p value** | **Adjusted* OR (95% CI)** | **p value** |  |
| **All** | 383,160,540 | 236,702 (0.11) |  |  |  |  |  |
| **Age** |  |  |  |  |  |  |  |
| 1-4 | 19,495,258 | 166,895 (0.86) | 10.65 (9.97, 11.36) | <.0001 | 10.93 (10.26, 11.65) | <.0001 |  |
| 5-9 | 13,711,004 | 25,909 (0.19) | 2.38 (2.24, 2.52) | <.0001 | 2.42 (2.28, 2.56) | <.0001 |  |
| 10-14 | 13,103,737 | 7,979 (0.06) | 1.00 |  | 1.00 |  |  |
| 15-19 | 20,351,087 | 7,657 (0.04) | 0.83 (0.78, 0.88) | <.0001 | 0.85 (0.80, 0.90) | <.0001 |  |
| 20-29 | 56,927,949 | 19,217 (0.03) | 0.74 (0.7, 0.79) | <.0001 | 0.78 (0.74, 0.83) | <.0001 |  |
| 30-39 | 55,146,446 | 20,889 (0.04) | 0.74 (0.69, 0.79) | <.0001 | 0.77 (0.72, 0.82) | <.0001 |  |
| 40-49 | 46,050,579 | 22,684 (0.05) | 0.77 (0.72, 0.81) | <.0001 | 0.79 (0.74, 0.84) | <.0001 |  |
| 50-59 | 48,955,014 | 38,801 (0.08) | 1.05 (0.99, 1.12) | <.0001 | 1.06 (0.99, 1.12) | 0.0752 |  |
| 60-69 | 44,607,702 | 56,872 (0.13) | 1.6 (1.5, 1.71) | <.0001 | 1.55 (1.46, 1.65) | <.0001 |  |
| >=70 | 64,811,764 | 113,645 (0.18) | 2.17 (2.03, 2.32) | <.0001 | 1.86 (1.75, 1.98) | <.0001 |  |
| **Sex** |  |  |  |  |  |  |  |
| Female | 209,363,286 | 236,702 (0.11) | 0.83 (0.81, 0.84) | <.0001 | 0.94 (0.92, 0.95) | <.0001 |  |
| Male | 173,797,254 | 243,849 (0.14) | 1.00 |  | 1.00 |  |  |
| **Race/Ethnicity** |  |  |  |  |  |  |  |
| White | 211,951,269 | 277,632 (0.13) | 1.00 |  | 1.00 |  |  |
| Black | 76,767,241 | 72,071 (0.09) | 0.73 (0.71, 0.76) | <.0001 | 0.75 (0.73, 0.78) | <.0001 |  |
| Hispanic | 60,536,001 | 71,381 (0.12) | 0.91 (0.87, 0.96) | 0.0003 | 0.78 (0.75, 0.81) | <.0001 |  |
| Asian | 8,738,802 | 17,786 (0.2) | 1.52 (1.42, 1.63) | <.0001 | 1.31 (1.23, 1.40) | <.0001 |  |
| Other | 25,167,227 | 41,680 (0.17) | 1.53 (1.29, 1.81) | <.0001 | 0.96 (0.85, 1.1) | 0.6366 |  |
| **Urbanicity** |  |  |  |  |  |  |  |
| Urban | 316,152,546 | 399,512 (0.13) | 1.06 (1, 1.12) | 0.0375 | 1.11 (1.06, 1.10) | <0.0001 |  |
| Suburban | 40,080,478 | 48,353 (0.12) | 1.01 (0.96, 1.07) | 0.7329 | 1.03 (0.98, 1.09) | 0.2339 |  |
| Rural | 26,927,516 | 32,686 (0.12) | 1.00 |  | 1.00 |  |  |
| **Payor** |  |  |  |  |  |  |  |
| Medicare/Medicaid | 209,566,802 | 314,872 (0.15) | 1.00 |  | 1.00 |  |  |
| Medicaid | 112,182,522 | 159,881 (0.14) |  |  |  |  |  |
| Medicare | 97,384,280 | 154,991 (0.16) |  |  |  |  |  |
| Private insurance | 111,521,543 | 122,291 (0.11) | 0.75 (0.73, 0.77) | <.0001 | 0.97 (0.94, 1.00) | 0.0348 |  |
| Self-pay | 44,107,749 | 27,175 (0.06) | 0.51 (0.48, 0.54) | <.0001 | 0.86 (0.82, 0.91) | <.0001 |  |
| Other | 17,964,447 | 16,213 (0.09) | 0.75 (0.71, 0.8) | <.0001 | 1.04 (0.98, 1.10) | 0.1770 |  |
| **Autism spectrum disorder** |  |  |  |  |  |  |  |
| Yes | 975,973 | 3,294 (0.34) | 2.69 (2.45, 2.95) | <.0001 | 1.60 (1.48, 1.74) | <.0001 |  |
| No | 382,184,567 | 477,257 (0.12) | 1.00 |  | 1.00 |  |  |
| **Intellectual disability** |  |  |  |  |  |  |  |
| Yes | 758,395 | 4,474 (0.59) | 4.48 (4.13, 4.87) | <.0001 | 5.65 (5.23, 6.11) | <.0001 |  |
| No | 382,402,145 | 476,077 (0.12) | 1.00 |  | 1.00 |  |  |
| **Attention-deficit hyperactivity disorder** |  |  |  |  |  |  |  |
| Yes | 2,626,302 | 1,877 (0.07) | 0.94 (0.86, 1.04) | <.0001 | 1.34 (1.23, 1.47) | <.0001 |  |
| No | 380,534,238 | 478,673 (0.13) | 1.00 |  | 1.00 |  |  |
| **Alzheimer's disease and related dementia** |  |  |  |  |  |  |  |
| Yes | 8,034,340 | 25,603 (0.32) | 2.39 (2.27, 2.53) | <.0001 | 2.08 (2.00, 2.17) | <.0001 |  |
| No | 375,126,200 | 454,947 (0.12) | 1.00 |  | 1.00 |  |  |
| *Adjusted for age, race, sex, urbanicity, payor, autism spectrum disorder, intellectual disability, attention-deficit hyperactivity disorder, and Alzheimer’s disease and related dementia. | | | | | | | |
